# Somatostatin receptor based hybrid imaging in sarcoidosis

**DOI:** 10.1186/s41824-017-0014-y

**Published:** 2017-12-15

**Authors:** Riemer H. J. A. Slart, Klaas-Pieter Koopmans, Peter Paul van Geel, Henk Kramer, Harry J. M. Groen, C. Tji-Joong Gan, Niek H. J. Prakken, Andor W. J. M. Glaudemans, George D. Nossent

**Affiliations:** 1 Medical Imaging Center (MIC), Department of Nuclear Medicine & Molecular Imaging and Radiology, University of Groningen, University Medical Center Groningen, Hanzeplein 1, P.O. Box 30001, 9700 RB Groningen, The Netherlands; 2 Department of Nuclear Medicine and Radiology, Martini Hospital, Groningen, The Netherlands; 3 Department of Cardiology, Groningen, The Netherlands; 4 Department of Pulmonology, Martini Hospital, Groningen, The Netherlands; 5 Department of Pulmonology, University of Groningen, University Medical Center Groningen, Groningen, The Netherlands

**Keywords:** Sarcoidosis, Cardiac, PET/CT, Somatostatin receptor imaging

## Abstract

Several diagnostic imaging methodologies are available for the clinical evaluation of sarcoidosis, but all have their limitations. FDG PET/CT is frequently used, but this technique does not provide optimal results in all cases. Novel radiopharmaceuticals aimed at other disease targets may be helpful, particularly in cardiac sarcoidosis when FDG PET/CT has a low diagnostic accuracy, due to difficulties in preparing the patients who should use a specific diet combined with prolonged fasting. ^68^Ga-labeled somatostatin based receptor hybrid imaging is a potential alternative to FDG PET/CT.

This short communication provides a rapid overview of initial findings concerning the application of ^68^Ga-labeled somatostatin based receptor hybrid imaging in the diagnosis of (cardiac) sarcoidosis activity.

## Introduction

Sarcoidosis is a granulomatous disorder of unknown etiology and is most commonly affecting lymph nodes in the mediastinum, hili and the parenchyma of the lungs, but it can involve any organ system (Baughman et al., 2001). Sarcoidosis affects approximately 10 out of 100,000 persons each year (Ungprasert et al., 2016). Cardiac sarcoidosis is reported to involve only 2% to 5% of patients with systemic sarcoidosis (Baughman et al., 2001; Lynch et al., 2014), but autopsy studies indicate a considerably higher prevalence of around 27% (Perry & Vuitch, 1995; Silverman et al., 1978).

The clinical presentation of cardiac sarcoidosis covers a large variability in arrhythmogenicity. This ranges from being asymptomatic to heart block, heart failure, complex arrhythmias and sudden cardiac death. The Heart Rhythm Society (HRS) has published an expert consensus statement which recommends that patients with sarcoidosis proved by biopsy and possible clinical cardiac manifestations should be screened for cardiac involvement (Birnie et al., 2014). Non-invasive, specific imaging is needed to evaluate the cardiac sarcoid involvement.

## Imaging

Several diagnostic imaging techniques for diagnosing sarcoidosis are available. Chest imaging plays a central role in the diagnosis and monitoring of sarcoidosis. Chest X-ray is usually the initial approach to evaluate the parenchymal and mediastinal and hilar lymph node involvement. High-resolution CT (HRCT) is more accurate in visualizing the various manifestations of pulmonary sarcoidosis, including its complications, but lacks specificity for mediastinal lymph node involvement. ^18^F–fluorodeoxyglucose positron emission tomography, combined with computed tomography (CT) (FDG PET/CT) is emerging as a useful adjunct in complicated cases of sarcoidosis, particularly for identifying occult (extra)thoracic disease activity, including targets for tissue diagnosis, and has also been used as a non-invasive marker of disease severity especially in Stage IV pulmonary fibrosis, therapy evaluation, and in cardiac sarcoidosis (CS). FDG PET/CT has been reported to display variable sensitivity and specificity for detecting CS, due to the absence of a specific diet and of prolonged fasting in some studies.(Youssef et al., 2012). Focal heterogeneous FDG uptake in the myocardium within the appropriate clinical context indicates cardiac involvement. The clinical application of FDG PET/CT to evaluate CS has several limitations. Myocardial FDG uptake can be highly variable and special preparation is required to suppress the glucose metabolism of the myocardial cells in order to eliminate physiological background myocardial FDG uptake as much as possible. Further pre-test optimization strategies are required, such as dietary modification or unfractionated heparin, as solely fasting may not be sufficient to suppress myocardial FDG uptake (Harisankar et al., 2011; Scholtens et al., 2016; Williams & Kolodny, 2008). FDG PET/CT may also show focal and heterogeneous uptake in the heart due to infarction or ischemia rather than CS, but in general, coronary artery disease is ruled out before by conventional diagnostics.

Specific radiopharmaceuticals without physiological myocardial background activity may be of additional value. ^68^Ga-labeled somatostatin based PET imaging is already available and used for other (oncological) applications, but may be promising to be applied in the diagnosis of (cardiac) sarcoidosis.

The somatostatin receptor subtype 2 (SSTR2) is highly expressed in sarcoid granulomas. This high SSTR2 receptor expression is beneficial for somatostatin receptor scintigraphy (SRS) using ^111^In-DTPA-D-Phe1-octreotide (Kamphuis et al., 2015). ^111^In-DTPA-D-Phe1-octreotide and several ^68^Gallium-labeled somatostatin analogues which are used for PET imaging possess a high SSTR2 affinity (Gormsen et al., 2016). ^68^Ga-DOTA-NaI-octreotide (DOTANOC), ^68^Ga-DOTA-_D_Phe(Baughman et al., 2001)-Tyr^3^-octreotate (DOTATATE) or ^68^Ga-DOTA-D-Phe-Tyr-octreotide (DOTATOC) could be used as alternatives to the non-specific tracer FDG in patients with suspected (cardiac) sarcoidosis. Epithelioid cells, giant cells, and macrophages that can be found in sarcoid granulomas highly express SSTRs (more specifically, SSTR2A) on their surface (ten Bokum et al., 1999). These somatostatin analogs have different affinities to the different subtypes of the SSTR receptor. ^68^Ga-DOTANOC has a high affinity to both SSTR2 and 5 (Prasad & Baum, 2010), whereas ^68^Ga-DOTATOC has mainly high affinity to SSTR2 (Nobashi et al., 2016). The main advantage of these tracers is the absence of uptake in the normal heart, due to the lack of SSTR2 expression on the myocardial cell. The resulting high signal-to-noise ratio is beneficial for ^68^Ga- SSTR2 imaging, allowing a more straightforward and reproducible image interpretation, even by less experienced readers/physicians as compared to FDG imaging. ^68^Ga- SSTR2 imaging also provides data on SSTR2 expression by all lesions of the individual patient (whole body composition), which can help in assessing whether treatment with cold or hot somatostatin analogs could be beneficial. Finally, the benefit of generator produced ^68^Ga isotopes bypasses the dependency of a cyclotron.

## Future

The Japanese Ministry of Health and Welfare (JMHW) has issued criteria for the diagnosis of sarcoidosis. However, these criteria do not mention SSTR2 PET imaging, and also exclude the use of FDG PET/CT in CS (Jpn Ministry Health Welfare, 2007). The HRS recommends FDG PET/CT as diagnostic in CS, but is not mentioning SSTR2 imaging. A low number of mainly small studies has reported on the use of SSTR2 PET/CT imaging in CS (Gormsen et al., 2016; Nobashi et al., 2016; Lapa et al., 2016). Larger, randomized prospective studies should confirm the value of SSTR2 PET/CT imaging, compared to conventional imaging such as cardiac FDG PET and cardiac magnetic resonance imaging (CMR).

More recent studies have highlighted the utility of integrated FDG PET/MRI in CS (Hanneman et al., 2017; Schneider et al., 2014; Wada et al., 2015). In general, the detection of pathologic glucose metabolism using FDG-PET could improve the specificity of CMR. Late gadolinium enhancement can be measured semi-quantitatively using contour tracing, and more specifically, T2 mapping can be performed to measure disease activity (edema) which can be used in therapy monitoring (Yang et al., 2014). T1 mapping (tissue characterization) can also quantify the amount of fibrosis and inflammation (edema) in CS (Puntmann et al., 2017). Combined FDG and CMR imaging with hybrid PET/MRI may be of value.

But does the same hold true for combined SSTR2 PET/MRI imaging? Future studies will be necessary to provide evidence for the clinical application of PET/MRI in CS (Nensa et al., 2015).

**Fig. 1:**
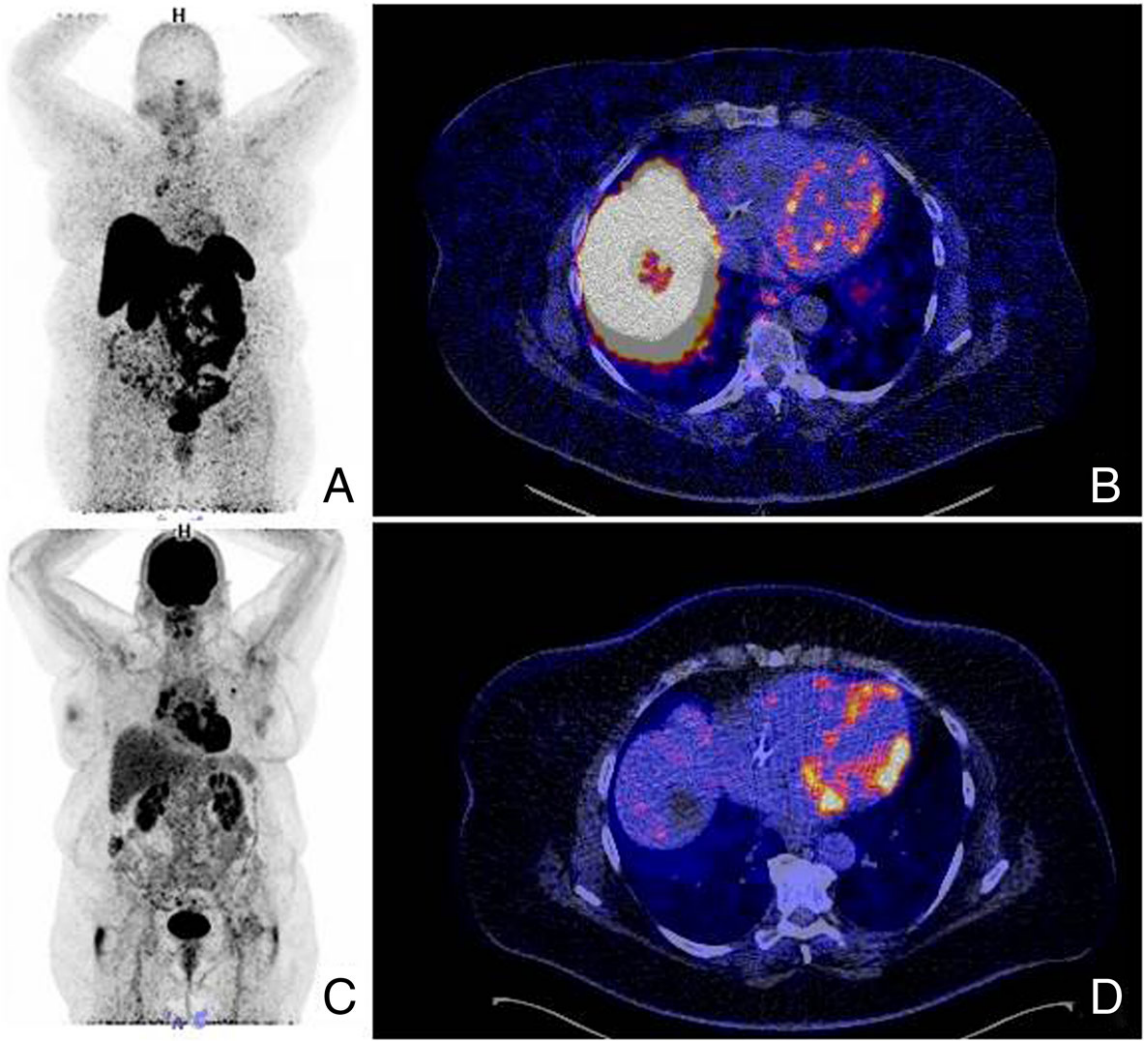
^68^Ga-Dotatoc whole body PET (**a**) in a 56-year old woman with chronic (cardiac) sarcoidosis. Increased uptake is visible in the lymph nodes located at the right-sideof the mediastinum and in lung hilar tissue. ^68^Ga-Dotatoc PET/CT uptake (**b**) is visible in the left ventricle of the heart (lateral, basal, septal) and the anterior part of the right ventricle. The same distribution pattern is visible on FDG whole body (**c**) and cardiac PET (**d**), but is more intense. Apparently FDG PET was more helpful than ^68^Ga-Dotatoc in the chronic phase of sarcoidosis in this patient

At the moment, it is unclear whether SSTR2 PET/CT imaging is more beneficial in acute or chronic sarcoidosis. The initial impression is that SSTR2 PET/CT imaging is most helpful in the acute phase of sarcoidosis. In the chronic phase, fibrotic tissue is formed. Fibrotic tissue lacks substantial SSTR2 expression, potentially resulting in a low signal in SSTR2 PET/CT (Nobashi et al., 2016). It’s questionable if SSTR2 PET/CT imaging will then be better as compared to FDG. FDG activity and SSTR expression are different in these phases. The most optimal approach is using FDG for acute inflammation at first diagnosis of CS or suspicion of recurrence or relapse, because of the impact on the therapeutic management. The chronic phase should be covered by the CMR. An example of this is shown in Fig. 1, of a patient familiar with general sarcoidosis, including cardiac involvement. PET/CT visualized high FDG uptake and moderate ^68^Ga-SSTR2 uptake. Further investigations are needed to assess in which phase of sarcoidosis SSTR2 PET/CT imaging should be applied.

Applying this ^68^Ga-SSTR2 in the chronic phase (relapse/recurrence) of (cardiac) sarcoidosis seems to be a potential role, maybe combined with CMR, using hybrid PET/MRI. Another option is application in patients that are unable/not willing to prepare for a FDG PET/CT scan by subjecting themselves to a low-carbohydrate/high fat diet and fasting regime.

Further, the expression of SSTR2 in sarcoid tissue can be used in the theranostic approach. High SSTR2 receptor expression suggests that somatostatin treatment may be an option in patients that are resistant to conventional treatment with corticosteroids. In these patients ^68^Ga-SSTR2 imaging might be helpful to evaluate and to quantify the SSTR2 expression and may predict the effect of treatment with somatostatin.

In conclusion*,* ^68^Ga labeled SSTR2 PET/CT seems an interesting alternative to FDG PET/CT in (cardiac) sarcoidosis, since these modalities have different imaging targets. Additional studies comparing these radiopharmaceuticals are needed, particularly to evaluate in which phase of sarcoidosis (acute or chronic) ^68^Ga labeled SSTR2 PET/CT will be more beneficial than FDG.
